# Exploring Risk of Falls and Dynamic Unbalance in Cerebellar Ataxia by Inertial Sensor Assessment

**DOI:** 10.3390/s19245571

**Published:** 2019-12-17

**Authors:** Pietro Caliandro, Carmela Conte, Chiara Iacovelli, Antonella Tatarelli, Stefano Filippo Castiglia, Giuseppe Reale, Mariano Serrao

**Affiliations:** 1 Unità Operativa Complessa Neurologia, Fondazione Policlinico Universitario A. Gemelli IRCCS, Largo A. Gemelli, 8, 00168 Rome, Italy; pietro.caliandro@policlinicogemelli.it; 2 IRCCS Fondazione Don Carlo Gnocchi, Piazzale Morandi, 6, 20121 Milan, Italy; cconte@dongnocchi.it; 3 Department of Occupational and Environmental Medicine, Epidemiology and Hygiene, INAIL, via Fontana Candida, 1, 00078 Monte Porzio Catone, Italy; antonellatatarelli@gmail.com; 4 Department of Medical and Surgical Sciences and Biotechnologies, Sapienza University of Rome, Piazzale Aldo Moro, 5, 00185 Rome, Italy; stefanofilippo.castiglia@uniroma1.it (S.F.C.); mariano.serrao@uniroma1.it (M.S.); 5 Department of Neurosciences, Università Cattolica del Sacro Cuore, Largo F. Vito, 1, 00168 Rome, Italy; giureale@yahoo.it; 6 Policlinico Italia, Movement Analysis Laboratory, Piazza del Campidano, 6, 00162 Rome, Italy

**Keywords:** inertial sensors, cerebellar ataxia, movement analysis, gait analysis, balance, personalized medicine, rehabilitation

## Abstract

Background. Patients suffering from cerebellar ataxia have extremely variable gait kinematic features. We investigated whether and how wearable inertial sensors can describe the gait kinematic features among ataxic patients. Methods. We enrolled 17 patients and 16 matched control subjects. We acquired data by means of an inertial sensor attached to an ergonomic belt around pelvis, which was connected to a portable computer via Bluetooth. Recordings of all the patients were obtained during overground walking. From the accelerometric data, we obtained the harmonic ratio (HR), i.e., a measure of the acceleration patterns, smoothness and rhythm, and the step length coefficient of variation (CV), which evaluates the variability of the gait cycle. Results. Compared to controls, patients had a lower HR, meaning a less harmonic and rhythmic acceleration pattern of the trunk, and a higher step length CV, indicating a more variable step length. Both HR and step length CV showed a high effect size in distinguishing patients and controls (p < 0.001 and p = 0.011, respectively). A positive correlation was found between the step length CV and both the number of falls (R = 0.672; p = 0.003) and the clinical severity (ICARS: R = 0.494; p = 0.044; SARA: R = 0.680; p = 0.003). Conclusion. These findings demonstrate that the use of inertial sensors is effective in evaluating gait and balance impairment among ataxic patients.

## 1. Introduction

Patients suffering from cerebellar ataxia exhibit peculiar spatiotemporal and kinematic features that contribute to an unstable gait [1,2,3,4,5]. The gait impairment typically worsens over time, in parallel with the functional decline associated to the neurodegenerative process [6,7]. While stable gait is characterized by repeatable walking patterns [8], steadiness in the case of perturbations [9,10,11,12,13], and effectiveness in maintaining upright balance [14,15], ataxic gait is extremely variable over gait cycles [1] and exhibits inefficient coordination between upper and lower segments of body, even in the absence of external perturbations [16]. Taking into account such conditions, it is reasonable to hypothesize that when perturbation occurs in ataxic patients, the consequent fall risk increases, and the gait pattern can be defined as unstable [6,7].

The evaluation of gait instability and fall risk is, therefore, pivotal in the study of ataxic gait to prevent further disabilities, and in order to maximize and optimize the information we gather from such evaluation, it should be performed in a real-life environment outside the motion analysis laboratory for a long period of time. In this context, wearable magnetic and inertial measurement units (MIMUs), consisting of a three-axial accelerometer, a gyroscope, and a magnetometer, represent a self-contained alternative to conventional laboratory-based motion capture systems [17,18,19]. This technology estimates the three-dimensional (3D) orientation of MIMUs with respect to a global coordinate system by specific sensor fusion algorithms, using angular velocity, gravity and magnetic field vectors.

A series of biomechanical stability measures based on MIMU evaluations have been proposed in several studies on neurological gait disorders with dynamic unbalance [3,20,21,22]. The maximum Lyapunov exponent (λmax) is an available method to evaluate gait instability [4] and fall risk [3] in ataxic patients, but the relationship between λmax and clinical severity has not been definitively established, since it has been demonstrated to be both positively [4] and negatively [3] correlated to International Cooperative Ataxia Rating Scale (ICARS) scores. A possible explanation could be found in the heterogeneous etiologies of the study samples, respectively acquired cerebellar lesions after tumor resection [4], and neurodegenerative ataxia [3]. Another important issue is that λmax properly explores the nonlinear dynamic local stability of the trunk during locomotion when at least 150 continuous strides are recorded [15]. However, such stride numbers are often not practically feasible in ataxic patients, and this could have influenced the correlation analysis between λmax and clinical severity. 

To the best of our knowledge, no other studies in the literature have used additional indexes of stability, like harmonic ratio (HR) and the coefficients of variation (CV) based on MIMU data to detect the instability of ataxic patients. Therefore, the aim of this study is to evaluate these indexes of stability and, in particular, examine the ability of each index to detect the instability of ataxic patients compared to healthy controls and determine the fall risk. HR was chosen to evaluate the trunk acceleration patterns, a key feature in determining the severity of the ataxic gait [5,16], while CV was chosen to evaluate the variability of step length, an important compensatory mechanism in ataxic patients.

## 2. Materials and Methods

### 2.1. Participants

Seventeen patients affected by primary degenerative cerebellar ataxia were enrolled in the study. Table 1 summarizes the patients’ clinical features and genotype. 

The complete neurological assessment included (1) cognitive evaluation according to mini-mental state examination (MMSE) scale, (2) cranial nerve evaluation, (3) muscle tone evaluation, (4) muscle strength evaluation, (5) joint coordination evaluation, (6) sensory examination, (7) tendon reflex elicitation, and (8) disease severity measured by International Cooperative Ataxia Rating Scale (ICARS) and Scale for the Assessment and Rating of Ataxia (SARA) [23,24]. We excluded patients with gait impairment due to extracerebellar symptoms or orthopedic disorders. Regarding the extracerebellar disorders affecting gait, we excluded patients with spasticity, polyneuropathy, cognitive deficits, and extrapyramidal disorders. Of the recruited patients, no one presented with signs of spasticity, hyposthenia, hypoesthesia, and/or cognitive impairment (MMSE > 24). All patients were able to walk alone without any kind of assistance or aid, and were receiving physical therapy, including active and passive exercises for upper and lower limbs as well as balance and gait re-education. Furthermore, no patient had significant visual deficits according to the Snellen visual acuity test. Almost all of the patients had non-disabling oculomotor abnormalities, such as nystagmus or square wave jerks pursuit movements, because of the underlying disorder. A brain MRI showed that all patients had cerebellar atrophy. Regarding the fall risk assessment, all patients had to complete a specific questionnaire designed to evaluate the number of falls in the previous year, the characteristics of such falls (side, associated injury), and the circumstances in which they occurred. The number of falls in the last year was used for correlation analysis. Sixteen age-matched healthy adults (age, ataxic patients 53.53 ± 12.12 years, healthy controls, 50.94 ± 8.79 years, p > 0.05) were enrolled as the control group. We obtained informed consent from each patient and healthy subject, which complied with the Helsinki Declaration and was approved by the local ethics committee.

### 2.2. Gait Analysis

We acquired data with an inertial sensor (BTS GWALK, BTS, Milan, Italy), attached to an ergonomic belt placed around the pelvis at the level of the L5 vertebra, connected to a portable computer via Bluetooth. The sampling rate was 100 Hz, and the sensor, endowed with a tri-axial accelerometer (16 bit/axes), a tri-axial magnetometer (13 bit), and a tri-axial gyroscope (16 bit/axes), measured the linear trunk accelerations and the trunk angular velocities in three space directions (i.e., AR: anterior-posterior; ML: mediolateral; VT: vertical direction).

### 2.3. Task Description

Before starting the experimental session, participants were asked to walk along a predetermined route in order to familiarize themselves with the procedure. Recordings of all the patients were obtained during overground walking. We asked participants to walk along a corridor (3 m wide and 20 m long) at their preferred speed. Control subjects were asked to walk at a low speed in order to match the two groups for speed (ataxic patients, 0.939 ± 0.195 m/s; controls, 0.924 ± 0.239 m/s; p > 0.05).

### 2.4. Inertial Sensor Data Processing

The ‘walking protocol’ of the inertial sensor (G-STUDIO, BTS, Milan, Italy) was used to detect: (1) trunk acceleration patterns, (2) right and left heel strikes, and (3) toe-off. The HR and the CV were calculated using MATLAB software (MATLAB 7.4.0, MAthWorks, Natick, MA, USA).

Harmonic ratio. The harmonic ratio (HR), initially described by Gage [25] and later modified by Smidt et al. [26], provides an indication of the acceleration patterns, smoothness, and rhythm. Since the unit of measurement from a continuous walking trial is a stride (two steps), a stable, rhythmic gait pattern should be characterized by multiples of two repeated acceleration patterns within any given stride. Accelerations patterns that do not repeat in multiples of two generate out of phase accelerations, reflecting irregular accelerations during a walking trial and, therefore, an unstable gait pattern. The harmonic content of the acceleration signals can be analyzed in each spatial direction using stride frequency as the fundamental frequency component. Based on each stride time, 20 harmonics were calculated. Trunk accelerations of each stride were broken down into individual sinusoidal waveforms using discrete Fourier transform (DFT).

Since a stable smooth gait pattern is characterized by acceleration signals in VT and AP directions that repeat in multiples of two during a single stride, HRs in the VT and AP directions were calculated as the ratio of the sum of the amplitudes of the first 10 even harmonics divided by the sum of the amplitudes of the first 10 odd harmonics. In the ML direction, acceleration signals were repeated once for any given stride, so HRs in the ML direction were calculated as the sum of the amplitudes of the odd harmonics divided by the sum of the amplitudes of the even harmonics. We used a high-pass filter with cutoff at 20 Hz to eliminate noise signals. 

HRs per stride were determined and averaged across a steady walk, resulting in a mean HR. HR in AP and VT, and in the ML direction, were calculated as below [19]:

HR in anterior–posterior and vertical directions

$$HR=\frac{\sum_{i=1}^{10}A_{2i}}{\sum_{i=1}^{10}A_{2i-1}}$$

HR in the medio-lateral direction

$$HR=\frac{\sum_{i=1}^{10}A_{2i-1}}{\sum_{i=1}^{10}A_{2i}}$$

where A_2i_ denotes the amplitude of the first 20 even harmonics and A_2i–1_ indicates the amplitude of the first 20 odd harmonics. The higher the HR value, the smoother the walking pattern.

Coefficient of variation. In order to compute the step length CV, the step length was estimated using the upward and downward movements of the trunk, as proposed by Zijlstra and Hof [27]. Assuming a compass gait type, the body’s center of mass (CoM) movements in the sagittal plane follow a circular trajectory during each single support phase. In this inverted pendulum model, changes in height of CoM depend on step length [27]. Thus, step length can be deduced by known height changes and predicted from geometrical characteristics as follows: $\mathrm{step}\;\mathrm{length}=2\sqrt{2\mathrm{lh}-h^{2}}$.

In this equation, h is equal to the change in height of the CoM, and l represents the pendulum length. Changes in vertical position were calculated by a double integration of the vertical acceleration. A high-pass filter (fourth-order zero-lag Butterworth filter at 0.1 Hz) was used in order to avoid integration drift. The difference between highest and lowest position during a step cycle was used to determine the amplitude of changes in the vertical position (h). Leg length was considered as pendulum length (l). Step length was calculated as the mean of step lengths observed during seven subsequent steps of each subject.

Then, the step length coefficient of variation (CV) was computed as follows: $CV=100\frac{SD}{mean}$ where mean is the mean step length and SD is the standard deviation over the entire step length for each subject [1]. The CV is a measure of the variability of a data set; the closer to 0 the CV is, the less variable the data are.

### 2.5. Statistical Analysis

We used the SPSS 17.0 software (SPSS Inc. Chicago, IL, USA) for statistical analysis. All data were expressed as mean ± standard deviation; p < 0.05 was considered statistically significant. We assessed the normality of distributions using the Shapiro-Wilk test.

Mean and standard deviation within subjects were computed for speed and stability indexes. We used the independent-samples t test to look for differences between the stability indexes of ataxic patients vs. controls. Cohen’s d index was used to assess the effect size of the stability indexes in the three spatial directions [28,29]. We used the Pearson’s test to investigate any correlation We used the Pearson test to investigate any correlation of acceleration HR and step length CV with (1) age, (2) height, (3) weight, (4) disease duration, (5) total ICARS and SARA scores and (6) number of falls in the last year.

## 3. Results

Looking at the low scores of ICARS and SARA, the recruited patients mainly showed cerebellar symptoms (see Table 1). 

HR in all three directions and step length CV were all significantly different when compared to the controls (Table 2). Briefly, the HR of patients was lower than the HR of healthy subjects, meaning a less harmonic and rhythmic acceleration pattern of the trunk, while the CV of step length was greater in patients than in the controls, indicating a more variable step length in ataxic patients. Both HR and CV of step length showed a high effect size in distinguishing patients and controls, but HR in all three directions showed a higher effect size score when compared to the CV (Table 2).

**Figure 1:**
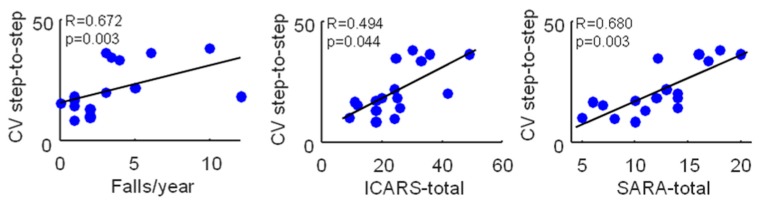
Correlations between the maximum step-to-step coefficient of variation and the falls/year, ICARS-total, and SARA-total scores in 17 ataxic patients. Pearson’s R coefficient (R) and significance (p) are reported.

Surprisingly, no correlation was found between HR in all directions, falls/year, and clinical severity (ICARS and SARA scores) (Table 3), while a significant positive correlation was found between the CV of step length and the falls/years and ICARS and SARA scores (Figure 1).

## 4. Discussion

In the present study, we found that trunk acceleration smoothness, as described by HR values, and the variability of step length, as described by the CV, may provide insights about gait stability in subjects with degenerative ataxia. Furthermore, the variability of step length correlated with both clinical severity and fall risk. 

Regarding the acceleration patterns of the trunk, the HR of patients significantly differed from that of healthy controls in all three spatial planes. Moreover, it showed a high effect size, according to Cohen’s d index (Table 2). This means that ataxic patients, compared to healthy subjects, exhibit a substantial reduction of trunk movement smoothness. When discussing these findings, we should bear in mind that the trunk has a great functional importance in minimizing the magnitude of linear and angular displacement of the head, ensuring clear vision [30,31], facilitating the integration of vestibular information [32], contributing to the maintenance of balance [5,6,16,33,34], and acting as a driving force for locomotion [35]. Consequently, investigating upper body stability in patients with degenerative cerebellar ataxia is essential, since the lack of motor control [5] and coordination [16] makes the trunk itself generate perturbations in a sort of vicious circle in parallel to the clinical decline [2]. In this context, trunk acceleration smoothness, as described by the HR values, provides a deeper insight into gait disturbances [14,36]. From the literature, we know that trunk acceleration smoothness during walking is predictive of gait dysfunction [37,38] and fall risk in older people [14,39]. Moreover, HR has already been found to be abnormal in patients who have suffered a stroke, Parkinson’s disease, or multiple sclerosis [19,20,22,40]. 

Overall, these findings suggest that HR can substantially describe trunk accelerative behavior abnormalities among patients with degenerative ataxia [41]. On the other hand, we did not find any correlation between HR, the number of falls, and clinical severity. This last result is apparently in contrast with previous studies that found a relationship between clinical severity, increased range of motion of trunk [5] and trunk–thigh coordination deficit [16]. Considering the small sample size of our study, we cannot exclude a type II error. Nevertheless, another possible explanation might come from the different implemented technologies and protocols. In fact, previous studies assessed the kinematic patterns of the upper segment of the head and the trunk via optoelectronic systems [5,16]. This means that the body markers were located on body segments (i.e., the head and upper trunk) whose range of movements was wider than the lumbar one, as investigated by a BTS GWALK device located on L5 vertebra. Further studies will assess such differences, evaluating the role of the ergonomic belt placed around the thorax just underneath the axilla, and will validate inertial sensor findings against optoelectronic systems.

The other parameter we considered was the CV of step length, which has been reported to be significantly different in subjects with cerebellar ataxia when compared to healthy subjects [42]. During the progression of the disease (i.e., >4 years from the onset, as in our sample), subjects with degenerative ataxia tend to lose the ability to both enlarge their step width and fasten their walking speed and—maintaining the same step width and speed—they shorten their step length in order to reduce their single support time [43], with a significant increase in step length CV that can lead to an increased risk of falls. In fact, we found that the CV of step length was higher in patients with ataxia than the controls and, unlike HR, the CV of step length significantly correlated with the ICARS and SARA scores and with the number of falls per year. These findings differ from those from a previous study, where a correlation between the CV of step length and clinical severity was not detected [1]. This difference might be due to both the use of different movement analysis technologies (inertial sensor vs. optoelectronic system) and different investigated samples (sporadic adult onset ataxia of unknown etiology (SAOA)/ spinocerebellar ataxia (SCA) vs. SCA/SAOA/Friedreich’s ataxia (FRDA)). Since a camera-based optoelectronic system can capture a smaller change of gait than MIMUs, our data should be interpreted with caution. However, the investigation of a large number of patients with FRDA in Serrao et al. [1] might explain, at least in part, such discrepant results. In this view, patients with FRDA and those with SCA and SAOA may show a different relationship between clinical features and gait stability; further studies are needed to explore this issue. However, our aim was not to obtain an alternative measure of step length, but to detect the relationship between the multifactorial gait impairment [5,6,16,33,34], clinical severity, and the fall risk. Because the MIMU-measured CV of step length is influenced by movements of the trunk [27], and trunk–thigh coordination is impaired in ataxic patients [16], our MIMU-measured CV might reflect trunk–thigh coordination variability. In this respect, the aforementioned limitation might come in handy, being such a multifactorial parameter able to summarize factors that, put together, explain gait instability in ataxic patients. 

Finally, our results cannot be generalized as representative of the ataxic population because they refer to patients with a disease duration of at least 8 years, preserved walking ability, and without extracerebellar symptoms as disabling oculomotor abnormalities. Moreover, our findings highlight the need to investigate the relationship between each MIMU-measured index and the corresponding ones measured by traditional optoelectronic systems in order to have proper validation.

## 5. Conclusions

In conclusion, the present study highlighted that both HR and CV differed between ataxic patients and healthy subjects. However, when considering the correlation with clinical severity and fall risk, only MIMU-measured CV of step length was able to describe the burden of ataxic symptoms and to draw clinical attention towards a possible increased fall risk. These MIMU-based parameters might provide real-world information on patients’ disabilities and falls, since they are obtained through wearable and comfortable devices.

## Figures and Tables

**Table 1:** Ataxic patients’ clinical and anthropometric characteristics.

	Number/Total	%	Mean (SD)
**Male**	9/17	52.9	-
**Female**	8/17	47.1	-
**Age (years)**	-	-	53.53 (12.12)
**Height (m)**	-	-	1.65 (0.09)
**Weight (kg)**	-	-	71.03 (12.74)
**ICARS**	-	-	24.70 (10.80)
**SARA**	-	-	12.20 (4.25)
**Disease duration (years)**	-	-	12.11 (4.52)
**Diagnosis**			
**SAOA**	9/17	52.9	-
**SCA1**	2/17	11.8	-
**SCA2**	3/17	17.6	-
**SCA3**	1/17	5.9	-
**SCA8**	1/17	5.9	-
**FRDA**	1/17	5.9	-

SAOA: sporadic adult onset ataxia of unknown etiology; SCA: spinocerebellar ataxia; FRDA: Friedreich’s ataxia.

**Table 2:** Comparisons of the stability indexes between 17 ataxic patients and 16 controls at matched gait speed.

Parameter	Patients	Controls	t	p	Cohen’s d
HR-AP	1.665 ± 0.300	2.414 ± 0.540	4.964	<0.001	1.714
HR-ML	1.639 ± 0.282	2.347 ± 0.559	4.631	<0.001	1.599
HR-VT	1.694 ± 0.304	2.549 ± 0.715	4.519	<0.001	1.556
Step length CV (%)	21.249 ± 10.293	13.205 ± 6.004	−2.720	0.011	0.955
Step length (m)	0.499 ± 0.087	0.569 ± 0.067	−2.382	0.024	0.112
Speed (m/s)	0.939 ± 0.195	0.924 ± 0.239	−0.207	0.838	0.069

Mean ± standard deviation values, the results of the independent samples t-test and Cohen’s d are reported. Values of p lower than 0.05 were considered statistically significant. HR-AP: harmonic ratio in the anterior–posterior direction; HR-ML: harmonic ratio in the mediolateral direction; and HR-VT: harmonic ratio in the vertical direction.

**Table 3:** Correlation analysis between HR in all directions and ICARS, SARA, and falls/year.

Parameter	ICARS (R, p)	SARA (R, p)	falls/year (R, p)
HR-AP	−0.35, 0.24	−0.35, 0.13	−0.10, 0.66
HR-ML	−0.47, 0.10	−0.36, 0.11	0.02, 0.92
HR-VT	−0.41, 0.88	−0.43, 0.06	−0.01, 0.99

The reported values represent Pearson correlation value (R) and statistical significance value (p). HR-AP: harmonic ratio in the anterior–posterior direction; HR-ML: harmonic ratio in the mediolateral direction; and HR-VT: harmonic ratio in the vertical direction.
